# Prehospital paths and hospital arrival time of patients with acute coronary syndrome or stroke, a prospective observational study

**DOI:** 10.1186/s12873-015-0065-y

**Published:** 2016-01-09

**Authors:** Carine J. M. Doggen, Marlies Zwerink, Hanneke M. Droste, Paul J. A. M. Brouwers, Gert K. van Houwelingen, Fred L. van Eenennaam, Rolf E. Egberink

**Affiliations:** Department of Health Technology and Services Research (HTSR), MIRA institute for Biomedical Technology and Technical Medicine, University of Twente, RA 5252, PO Box 217, 7500 AE Enschede, The Netherlands; Department of Neurology, Medisch Spectrum Twente, Enschede, The Netherlands; Department of Cardiology, Thoraxcentrum Twente, Medisch Spectrum Twente, Enschede, The Netherlands; Ambulance Oost, Hengelo, The Netherlands; Regional Network for Emergency Care, Acute Zorg Euregio, Enschede, The Netherlands

**Keywords:** Acute stroke, Prehospital paths, Delay, Acute coronary syndrome

## Abstract

**Background:**

Patients with a presumed diagnosis of acute coronary syndrome (ACS) or stroke may have had contact with several healthcare providers prior to hospital arrival. The aim of this study was to describe the various prehospital paths and the effect on time delays of patients with ACS or stroke.

**Methods:**

This prospective observational study included patients with presumed ACS or stroke who may choose to contact four different types of health care providers. Questionnaires were completed by patients, general practitioners (GP), GP cooperatives, ambulance services and emergency departments (ED). Additional data were retrieved from hospital registries.

**Results:**

Two hundred two ACS patients arrived at the hospital by 15 different paths and 243 stroke patients by ten different paths. Often several healthcare providers were involved (60.8 % ACS, 95.1 % stroke). Almost half of all patients first contacted their GP (47.5 % ACS, 49.4 % stroke). Some prehospital paths were more frequently used, e.g. GP (cooperative) and ambulance in ACS, and GP or ambulance and ED in stroke. In 65 % of all events an ambulance was involved. Median time between start of symptoms and hospital arrival for ACS patients was over 6 h and for stroke patients 4 h. Of ACS patients 47.7 % waited more than 4 h before seeking medical advice compared to 31.6 % of stroke patients. Median time between seeking medical advice to arrival at hospital was shortest in paths involving the ambulance only (60 min ACS, 54 min stroke) or in combination with another healthcare provider (80 to 100 min ACS, 99 to 106 min stroke).

**Conclusions:**

Prehospital paths through which patients arrived in hospital are numerous and often complex, and various time delays occurred. Delays depend on the entry point of the health care system, and dialing the emergency number seems to be the best choice. Since reducing patient delay is difficult and noticeable differences exist between various prehospital paths, further research into reasons for these different entry choices may yield possibilities to optimize paths and reduce overall time delay.

## Background

In myocardial infarction (MI), percutaneous coronary intervention (PCI) is the treatment of choice usually followed by stent implantation. Treatment of ischemic stroke consists of intravenous thrombolysis with recombinant tissue plasminogen activator (rt-PA). In both cases treatment should start as soon as possible after first symptoms to prevent further tissue damage. According to guidelines, primary PCI should preferably start within 90 min after first medical contact for patients with ST-elevated myocardial infarction [1]. Most stroke guidelines recommend treatment with rt-PA within 4.5 h after first symptoms [2, 3]. In line with these guidelines, sets of quality indicators have been developed. Door-to-balloon time indicates the timeframe between arrival of the patient with myocardial infarction at the hospital and start of PCI. Door-to-needle time indicates the time between arrival of patient with ischemic stroke at the hospital and start of thrombolysis. However, mainly because of prehospital delay, many patients arrive too late for treatment, and in clinical reality across the entire stroke population this treatment can be given to only a minority (1–8 %) of such patients [4]. Improving these disappointing numbers seems to be very difficult.

Prior to arrival at the hospital patients may have had contact with several healthcare providers. In the Netherlands most patients contact their general practitioner (GP), and outside office hours most patients contact a GP cooperative. Alternatively, patients dial the national emergency number, and the dispatch center will send an ambulance if requested and warranted according to a standard protocol. Others will directly visit the Emergency Department (ED) of the hospital. This points out that several healthcare providers, and thus various prehospital paths may be involved.

Information about various prehospital paths and associated time delays is scarce [5, 6]. Therefore, the aim of this study was to describe the prehospital paths of patients with a presumed diagnosis of acute coronary syndrome or stroke and measure time to hospital treatment. Additionally, door-to-balloon time of patients with ST-elevated myocardial infarction and door-to-needle time of patients with ischemic stroke were assessed.

## Methods

### Study design and study population

This is a prospective observational study. Patients with a provisional diagnosis of Acute Coronary Syndrome (ACS) or stroke, suggested by the health care provider who was contacted by the patient in the prehospital phase, were included between May 2012 and July 2012 and between September 2012 and October 2012. Patients were 18 years or older and living in the region of Twente and Oost Achterhoek, the Netherlands. About 750,000 inhabitants live in this area of 2093 km^2^ where three hospitals, four GP cooperatives and two ambulance emergency medical services are active. No other in- or exclusion criteria were used.

Patients with a provisional diagnosis of ACS were hospitalized at a coronary care unit (CCU) in one of the three hospitals. One of these hospitals has the facilities to perform percutaneous coronary interventions (PCI). If a ST-elevated myocardial infarction was suspected based on the electrocardiogram (ECG) made in the ambulance, patients were transported to the hospital with PCI facilities; otherwise the patient was transported to the nearest hospital. Patients with a provisional diagnosis of stroke were transported to the nearest hospital. All three hospitals have a fully equipped stroke unit with facilities to provide thrombolysis.

### Data collection

Nurses(specialists) at the CCU or stroke unit informed (both orally and written) and asked admitted patients to participate in the study within 24 h of arrival. Each participating patient provided written informed consent and filled in a questionnaire, sometimes with some help of a relative. This structured questionnaire included questions regarding date, time, circumstances of the ACS or stroke, date and time of seeking medical advice, and the healthcare provider(s) the patient had contact with. The ambulance emergency medical services, EDs of the three hospitals, individual GPs and GP Cooperatives received an electronic questionnaire, focusing on date, time of telephone call from the patient or others, and date and time of visit when applicable. Data on arrival date and time at CCU or stroke unit, time of PCI or thrombolysis, and final diagnoses at hospital discharge were retrieved from hospital registries. In the few cases where similar data was asked for but different answers were obtained, data from health care providers and hospital registries were used. The study was submitted to the accredited medical ethical committee Twente and was deemed to be non-intrusive and therefore did not fall under the Dutch law governing scientific research with humans.

Patient delay is defined as the difference between the time of onset of symptoms according to the patient and the time the patient, family member, friend or bystander decided to call or visit a healthcare provider (first medical contact where medical advice is given). Furthermore, time between first medical contact and arrival at CCU (ACS patients) or ED (stroke patients) is calculated. Additionally, time between onset of symptoms and arrival at hospital is calculated. No upper time limits were used. Door-to-balloon time for patients receiving PCI is defined as the difference between time at arrival in hospital and starting time of the PCI procedure. Since most patients in need for PCI are transported directly to the CCU, door-time of CCU is used. For those patients who arrived at the ED, arrival time at ED is used as door-time. Door-to-needle time for stroke patients receiving thrombolysis is defined as the difference between arrival time at the ED and start of thrombolysis.

### Data analyses

For continuous variables means and standard deviations (SD) were calculated for normally distributed data. Median values with interquartile ranges (IQR 25-75^th^ percentiles) were calculated for non-parametric continuous data. Categorical variables are presented as absolute values and percentages.

## Results

Included in the study were 202 patients with a provisional diagnosis of acute coronary syndrome and 243 patients with a provisional diagnosis of stroke. Most patients with acute coronary syndrome were men (65.8 %) and the mean age was 63.3 years (Table 1). Final diagnoses of these 202 patients were: 19.3 % ST-elevated myocardial infarction, 20.8 % non ST-elevated myocardial infarction, 22.8 % unstable angina pectoris, 27.7 % aspecific thoracic complaints and 9.4 % other diagnoses, such as intercostal myalgia or atrial fibrillation. Half of the 243 patients with presumed stroke were men (50.2 %) and mean age was 69.4 years. Four patients were hospitalized twice during the inclusion period. Overall 74.9 % of the patients were finally diagnosed with acute ischemic stroke, 7.4 % transient ischemic attack, 7.8 % hemorrhagic stroke and 9.9 % with other diagnoses, such as peripheral vertigo or epileptic seizure.

**Table 1:** Characteristics and circumstances of 202 patients with ACS and 243 patients with stroke

	ACS (*N* = 202)	Stroke (*N* = 243)
	*N* (%)^b^	*N* (%)^b^
Men	133 (65.8)	122 (50.2)
Age, mean (SD)	63.3 (13.1)	69.4 (12.9)
Location when having symptoms^a^		
At home	146 (72.3)	188 (77.7)
Public place	47 (23.3)	39 (16.1)
Other	9 (4.5)	15 (6.2)
Other person present when having symptoms^a^		
Nobody	59 (29.2)	69 (28.6)
Partner	110 (54.5)	116 (48.1)
Family, friends or acquaintances	23 (11.4)	35 (14.5)
Other	10 (5.0)	21 (8.7)
Person who sought medical advice^a^		
Patient	112 (57.4)	69 (28.4)
Partner	47 (24.1)	80 (32.9)
Family, friends or acquaintances	17 (8.7)	57 (23.5)
Other	19 (9.7)	37 (15.2)

^a^≤ two missings

^b^Number and percentage, unless otherwise stated

About three-quarter of the patients were at home when symptoms started and in half of the events their partner was present. In the ACS group 57.4 %, and in the stroke group 28.4 % sought medical advice themselves.

### Prehospital paths

**Fig. 1:**
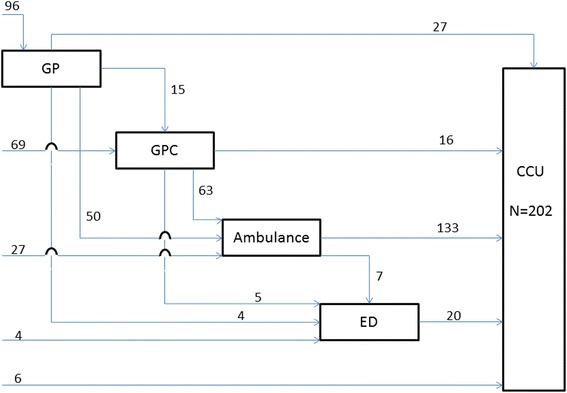
Prehospital paths of 202 patients with ACS

The 202 patients of the ACS group arrived at the CCU by 15 different paths (Fig. 1). In the prehospital phase 39.2 % had contact with one healthcare provider and 60.8 % with two or more health care providers. The four most frequently used prehospital paths were ‘GP cooperative and ambulance’, ‘GP and ambulance’, ‘GP only’ and ‘ambulance only’ (Table 2). Of the 202 patients 96 (47.5 %) had first medical contact with their GP and 69 (34.2 %) with the GP cooperative. Only 27 (13.4 %) patients immediately called the national emergency number. Of the 96 patients who first had contact with their GP, 50 (52.1 %) were transported by ambulance, and 27 (28.1 %) arrived at the CCU using private transportation. Of the 69 patients who first had contact with a GP cooperative, 51 (73.9 %) were transported by ambulance, and 12 (17.4 %) arrived at the CCU by private transportation. Overall, ambulances transported 140 out of 202 (69.0 %) patients with ACS of whom 133 directly to the CCU.

**Table 2:** Time (minutes) between onset of symptoms, seeking medical advice and arrival at CCU for patients with ACS overall and by most common prehospital paths

	Overall	Most common prehospital path			
		GP cooperative – ambulance	GP – ambulance	GP only	Ambulance only
	*N* = 202	*N* = 51 (25.2 %)	*N* = 49 (24.3 %)	*N* = 27 (13.4 %)	*N* = 25 (12.4 %)
	*N* (%)^a^	*N* (%)^b^	*N* (%)^b^	*N* (%)^b^	*N* (%)^b^
Symptom - arrival					
Median (25-75^th^)	385 (130–2859)	172 (99–608)	582 (155–4285)	4333 (416–13,184)	120 (82–405)
< 90	24 (12.1)	7 (13.7)	3 (6.3)	2 (7.7)	6 (26.1)
90-239	61 (30.8)	23 (45.1)	15 (31.3)	3 (11.5)	10 (43.5)
240-359	8 (4.0)	3 (5.9)	3 (6.3)	1 (3.8)	0 (0.0)
> = 360	105 (53.0)	18 (35.3)	27 (56.3)	20 (76.9)	7 (30.4)
Symptom - medical advice^c^					
Median (25-75^th^)	180 (30–1425)	95 (20–360)	495 (60–4085)	1290 (53–13,020)	60 (30–353)
< 90	82 (41.2)	24 (47.1)	16 (34.0)	7 (26.9)	16 (64.0)
90-239	22 (11.1)	10 (19.6)	4 (8.5)	0 (0.0)	2 (8.0)
240-359	14 (7.0)	4 (7.8)	2 (4.2)	1 (3.8)	1 (4.0)
> = 360	81 (40.7)	13 (25.5)	25 (53.2)	18 (69.2)	6 (24.0)
Medical advice - arrival					
Median (25-75^th^)	95 (60–196)	80 (60–113)	100 (71–167)	197 (100–1678)	60 (45–74)
< 90	91 (46.0)	29 (56.9)	19 (39.6)	6 (23.1)	20 (87.0)
90–239	65 (32.8)	15 (29.4)	20 (41.7)	9 (34.6)	3 (13.0)
240–359	6 (3.0)	1 (2.0)	1 (2.1)	1 (3.8)	0 (0.0)
> = 360	36 (18.2)	6 (11.8)	8 (16.7)	10 (38.5)	0 (0.0)

^a^≤ four missings

^b^≤ two missings

^c^Is patient delay

**Fig. 2:**
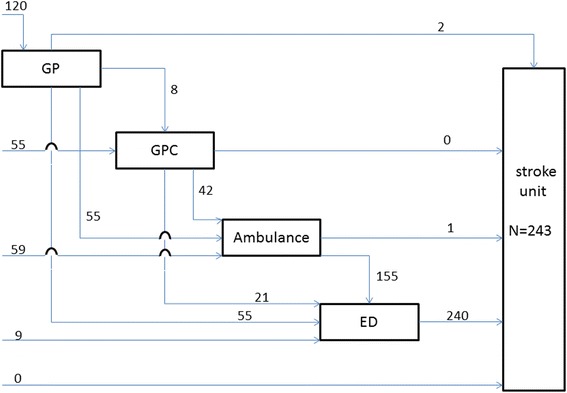
Prehospital paths of 243 patients with stroke

Patients in the stroke group arrived at the stroke unit through 10 different paths (Fig. 2). Almost five percent (4.9 %) of the 243 patients had contact with only one healthcare provider and 95.1 % with two or more health care providers before arriving at the stroke unit. The four most frequently used prehospital paths were ‘ambulance and ED’, ‘GP and ED’, ‘GP, ambulance and ED’ and ‘GP cooperative, ambulance and ED’ (Table 3). Of the 243 patients 120 (49.4 %) first sought medical advice from their GP, 55 (22.6 %) patients contacted the GP cooperative first, and 59 (24.3 %) patients immediately called the national emergency number. Almost half of the patients who first had contact with their GP were transported by ambulance (45.8 %) and others arrived at the ED using private transportation (45.8 %). Of the 55 patients who first contacted the GP cooperative 35 (63.6 %) were transported by ambulance and 20 (36.4 %) arrived at the ED by their own means. Overall, the ambulances transported 156 out of 243 patients (64.0 %) with a stroke.

**Table 3:** Time (minutes) between onset of symptoms, seeking medical advice and arrival at ED for patients with stroke overall and by most common prehospital paths

	Overall	Most common prehospital path			
		Ambulance – ED	GP – ED	GP – ambulance – ED	GP cooperative – ambulance – ED
	*N* = 243	*N* = 58 (23.9 %)	*N* = 55 (22.6 %)	*N* = 55 (22.6 %)	*N* = 35 (14.4 %)
	*N* (%)^a^	*N* (%)^b^	*N* (%)^b^	*N* (%)^b^	*N* (%)^b^
Symptom – arrival					
Median (25-75^th^)	240 (90–1031)	83 (49–288)	781 (291–1929)	209 (98–1031)	183 (83–1206)
< 60	34 (14.6)	21 (38.2)	1 (1.9)	6 (11.5)	5 (14.3)
60–239	82 (35.3)	17 (31.0)	9 (17.1)	23 (44.2)	18 (51.4)
240–359	24 (10.3)	6 (10.9)	8 (15.1)	5 (9.6)	2 (5.8)
> = 360	92 (39.7)	11 (20.0)	35 (66.0)	18 (34.6)	10 (28.6)
Symptom – medical advice^c^					
Median (25–75^th^)	60 (10–450)	15 (5–60)	195 (60–1140)	60 (15–315)	23 (5–300)
< 60	117 (48.8)	42 (73.7)	13 (23.6)	25 (46.3)	22 (64.7)
60–239	47 (19.7)	6 (10.5)	16 (29.1)	13 (24.1)	4 (11.8)
240–359	7 (2.9)	2 (3.5)	2 (3.6)	2 (3.7)	0 (0)
> = 360	69 (28.7)	7 (12.3)	24 (43.6)	14 (25.9)	8 (23.5)
Medical advice – arrival					
Median (25–75^th^)	98 (54–217)	54 (35–93)	238 (111–402)	106 (59–266)	99 (64–174)
< 60	62 (27.7)	28 (51.9)	4 (8.0)	12 (24.0)	7 (20.6)
60–239	111 (49.5)	21 (38.9)	22 (44.0)	25 (50.0)	23 (67.6)
240–359	18 (8.1)	1 (1.9)	9 (18.0)	6 (12.0)	1 (2.9)
> = 360	33 (14.7)	4 (7.4)	15 (30.0)	7 (14.0)	3 (8.8)

^a^≤ 19 missings

^b^≤ five missings

^c^Is patient delay

### Time delays

Median time between onset of symptoms and time to arrival at CCU in the ACS group was 385 min. Median patient delay was 180 min and 47.7 % of all the patients waited more than 4 h before seeking medical advice (Table 2). After first medical contact, 46 % of the patients arrived within 90 min at the CCU. Of the most common prehospital paths, median time between seeking medical advice and arrival at the CCU was shortest for ‘ambulance only’ (60 min). In line with this, the percentage of patients arriving at CCU within 90 min after first medical contact was highest for ‘ambulance only’ (87.0 %) and lowest for ‘GP only’ (23.1 %).

Median time between onset of symptoms and time to arrival at ED in the stroke group was 240 min. Median patient delay was 60 min and 31.6 % waited longer than 4 h before seeking medical advice (Table 3). After first medical symptoms, 49.9 % of the patients arrived within 4 h at the ED. Median time between seeking medical advice and arrival at ED was shortest for ‘ambulance and ED’ (54 min). Of patients using the path ‘ambulance and ED’ 69.2 % arrived within 4 h at ED after onset of symptoms and only 19.0 % of those using ‘GP and ED’.

### Door-to-balloon time and door-to-needle time

Overall 39 patients had a ST-elevated myocardial infarction. Six patients called or visited a healthcare provider more than 12 h after the first symptoms. ECG and clinical criteria of two patients were insufficient to perform PCI. One patient received a PCI in another hospital outside the region and details of the PCI were unknown. Median time between first medical contact and PCI for the remaining 30 patients with ST-elevated myocardial infarction was 132 min. Only one patient received a PCI within 90 min after first medical contact and 12 within 120 min (Table 4). Median door-to-balloon time was 50 min and 63.0 % received a PCI within a door-to-balloon time of 90 min.

**Table 4:** Time between onset of symptoms, seeking medical advice, PCI and door-to-balloon time for 30 patients with ST-elevated MI, thrombolysis and door-to-needle time for 31 patients with ischemic stroke

30 patients with ST-elevated MI who received percutaneous coronary intervention (PCI)				
Time (minutes)	Symptom – medical advice^a,b^	Medical advice – PCI	Symptom – PCI	Door-to-balloon^b^
	*N* (%)	*N* (%)	*N* (%)	*N* (%)
Median (25–75^th^)	30 (15–180)	132 (108–209)	222 (139–416)	50 (33–150)
< 60	17 (63.0)	0 (0.0)	0 (0.0)	14 (51.9)
60–89	2 (7.4)	1 (3.3)	0 (0.0)	3 (11.1)
90–119	0 (0.0)	11 (36.7)	4 (13.3)	2 (7.4)
120–239	2 (7.4)	12 (40.0)	14 (46.7)	6 (22.2)
240–359	2 (7.4)	4 (13.3)	2 (6.7)	1 (3.7)
> = 360	4 (14.8)	2 (6.7)	10 (33.3)	1 (3.7)
31 patients with ischemic stroke who received thrombolysis				
Time (minutes)	Symptom – medical advice^a,b^	Medical advice – thrombolysis^b^	Symptom – thrombolysis	Door-to-needle^b^
	*N* (%)	*N* (%)	*N* (%)	*N* (%)
Median (25–75^th^)	10 (5–30)	92 (64–120)	110 (77–150)	43 (33–53)
< 60	26 (86.7)	7 (23.3)	3 (9.7)	26 (89.6)
60–89	3 (10.0)	7 (23.3)	9 (29.0)	1 (3.4)
90–119	0 (0.0)	8 (26.7)	6 (19.3)	0 (0.0)
120–269	1 (3.3)	7 (23.3)	11 (35.5)	1 (3.4)
270–359	0 (0.0)	1 (3.3)	2 (6.5)	0 (0.0)
> = 360	0 (0.0)	0 (0.0)	0 (0.0)	1 (3.4)

^a^Patient delay

^b^≤ three missings

Out of 182 patients with ischemic stroke, 31 (17.0 %) received thrombolysis. Median time between first medical contact and thrombolysis was 92 min. Almost all patients (93.5 %) received thrombolysis within 270 min (4.5 h) after symptoms (Table 4). Median door-to-needle time was 43 min, and 89.6 % received thrombolysis within 60 min after arrival in hospital. Of the 151 patients not receiving thrombolysis 89 (58.9 %) arrived at the ED more than 4 h after the start of the symptoms and were therefore too late for thrombolysis. Although 62 (41.1 %) patients arrived within 4 h after the symptoms were first noticed, 24 of these patients could not receive thrombolytic treatment because the symptoms were first noticed on awakening, and therefore the precise starting time of stroke was unknown. The other 38 patients had contra-indications for thrombolysis such as the use of oral anticoagulants, recent surgery, minor neurological deficit and loss of consciousness.

## Discussion

Patients in the ACS group arrived at the CCU through 15 different paths, and patients in the stroke group arrived at the stroke unit through 10 different paths. In these paths often two or more healthcare providers were involved (60.8 % ACS, 95.1 % stroke). Almost half of all patients first contacted their GP (47.5 % ACS, 49.4 % stroke). Some prehospital paths were more frequently used, and in 65 % of all events an ambulance was involved, which is much higher compared to other countries [7]. For paths involving the ambulance either alone or in combination with one other healthcare provider, time intervals were short. Time intervals of other paths without ambulances were longer and differed considerably in duration. The major part of the overall prehospital delay was caused by patients themselves, as they waited a long time before seeking medical advice.

### Prehospital paths and time delays

With various prehospital paths through which patients arrived in the hospital various time delays occurred. Longer time delays occur when more health care providers are involved. When (only) an ambulance is involved, paths and delays are shorter, as shown in other studies [7–9]. If a ST-elevated myocardial infarction is suspected based on the ECG made in the ambulance or if the patient has typical symptoms, a phone call to the hospital with PCI facilities is made, the ECG is digitally send, and the patient is directly transported to the cardiac catheterization laboratory. As a result, the percentage of ACS patients arriving at CCU within 90 min after first medical contact was highest (87 %) when patients were directly transported by ambulance. Within these 90 min lies the optimal time to perform a PCI for patients with ST-elevated myocardial infarction [1].

Similarly, 69 % of all stroke patients who were directly transported by ambulance arrived within 4 h after onset of symptoms at the ED. The overall time window for rt-PA treatment is 4.5 h [2]. Before rt-PA can be delivered, a diagnostic work-up, including neurological examination, imaging and laboratory analysis, is necessary to exclude hemorrhage and diseases mimicking stroke, and to identify other contraindications. Moreover the diagnosis of ischemic stroke needs to be confirmed. Therefore, patients who arrive more than 4 h after the start of the symptoms are too late to be treated with thrombolytic therapy.

The challenge is to identify the patients that will benefit from a fast path by ambulance to the hospital. National protocols for ambulance services exist. However, the correct identification of stroke symptoms is not easy; on the one hand, symptoms can be difficult to recognize and on the other hand, as many as 20 % of presumed stroke symptoms are caused by completely different diseases. The proportion of strokes correctly identified by emergency medical systems dispatchers varies between 45 % and 83 % pointing strongly in the direction of continuous medical educational programs to improve on stroke recognition [10–13]. Another challenge is to educate patients to recognize ACS and stroke, and then call the national emergency number immediately.

### Contact with GP

Although patients arrived at the hospital through various prehospital paths, almost half contacted their own GP first. This is far less than in 1998–1999 when 87 % first contacted their GP [8]. In the Netherlands this is usual care, GPs act as gate keepers for hospitals. On the other hand, GPs may receive many patients with complaints which at first sight point in the direction of an ACS or stroke, but which after history taking and physical examination appear to be another disease. This study does not have insight into the number of patients who present themselves to the GP but are not transferred to the hospital. Nevertheless, where the GP was involved or where private transportation was used, time intervals were rather long, as reported before [8, 14]. Reasons for these long time intervals are unclear. It might be that more patients visiting their GP have aspecific symptoms which makes it difficult to ascertain a diagnosis and act quickly. Delays in women may be specifically long as they appear to have aspecific symptoms of myocardial infarction; although findings regarding differences by gender are inconsistent [15, 16]. In contrast to myocardial infarction, classic stroke symptoms do not differ between men and women [17].

This study showed that time intervals of prehospital paths involving a GP cooperative were shorter than when a patients’ own GP was involved. Since during office hours patients have to go to their own GP and during the evening, night or in the weekend to a GP cooperative, both organizations are likely to receive similar patients. What may explain differences in time intervals is the way both are organized (e.g. having a direct phone line to ambulance emergency medical services, different triage systems); although other reasons such as patients postponing seeking medical advice until Monday morning when their GP is available, or patients visiting GP cooperative with more serious symptoms, cannot be ruled out.

Almost half of the patients who presented themselves to their GP were subsequently transported by ambulance to the hospital. This indicates that some patients, in terms of time delay, could have benefited from bypassing the GP by calling the national emergency number instead. This confirms that the use of emergency medical systems is crucial to reduce prehospital delay [14]. The difference in contacting the ambulance between GP (~50 %) and GP cooperative (80 % ACS, 64 % stroke) is striking. Further research may give insight into reasons for this difference. Additionally, a study might investigate the effect of educating GPs in choosing the most efficient prehospital path.

There were some strange observations at first sight. A few patients had contact with their own GP and later on with a GP cooperative. Obviously, the provisional diagnosis of ACS or stroke was not made at first visit to the GP. Other reasons were not specifically investigated but this finding adds to the complexity of the prehospital routing of patients. Another finding was that not every patient with a provisional diagnosis of ACS or stroke was transported with an ambulance according to the guidelines. When we looked into this we found many good reasons not to call for an ambulance. For example, 20 out of 27 ACS patients who contacted their own GP first and went to the CCU with private transportation waited more than 6 h (some even days) before seeking medical advice and some patients simply refused to be transported by ambulance (personal communication). These findings add to the complexity of real world prehospital paths.

### Patient delay

The prehospital delay strongly depends on the choice of the patient when and where to enter the health care system. It is of interest to know what causes specific care-seeking behavior in patients. Which patients chose to contact their GP, GP cooperative or immediately called the emergency number? This knowledge may lead to more focused interventions in reducing delays. Most of the delay in the overall prehospital paths was due to the patient who waits too long before contacting a health care provider. Although three-quarter of the patients were at home and half of the patients were in the company of a partner when symptoms started, patient delay was long. Almost half of all ACS patients waited more than 4 h before seeking medical advice compared to over 30 % of all stroke patients. Patient delay is a worldwide problem and demographic, social, cognitive and emotional factors, as well as clinical characteristics play a role [18]. Median delays by ACS patients in the US vary between 1.5 and 6 h [18] compared to 3 h in this study. For stroke patients median delays differ from 53 min to 2 h in different studies [19–21]. Median time in the present study was one hour, meaning that 50 % of all patients waited longer than one hour before seeking medical advice. Apparently, patient delay did not change over the last 14 years in the Netherlands [8]. In contrast, overall median time between onset of symptoms and arrival at hospital seems to be reduced, from 5 h and 10 min in 1998–1999 to 4 h in our study. Minimizing patient delay in seeking care in ACS or stroke patients by mass media interventions is disappointing. Public health campaigns aiming to recognize symptoms and to get professional help as early as possible, are marginally successful, last only a few months, or do not influence patient delay at all [7, 22–24].

### Door-to-balloon time and door-to-needle time

Although door-to-balloon time for patients with ST-elevated myocardial infarction in need of PCI and door-to-needle time in stroke patients may be improved, in-hospital delays are very small compared to the overall time between onset of symptoms and arrival at hospital. Obviously, door-to-balloon time and door-to-needle time protocols have proven their value. Much more time is to gain in the prehospital phase.

### Strengths and limitations

One of the strengths of this study is that data was provided by patients themselves using structured questionnaires together with data extracted from registries. This gives an entire overview of the numerous and complex prehospital paths, which, to our knowledge, has been investigated in only a few studies and in the Dutch situation never to such an extent. The study is limited by a low consecutive recruitment rate. About 21 % of all ACS patients and 37 % stroke patients participated. We did not keep record of the reasons for exclusion. Therefore we do not know the reason for non-participation of each specific patient. During a discussion of the results with healthcare professionals from CCUs and stroke units, it appeared that refusal of the patient was rarely the reason for non-participation. In reality the attending nurse(specialist)s were too busy and forgot to ask patients to participate. We think that it is very unlikely that these reasons are associated with the various pre-hospital paths. The design of the study precluded patients who died before reaching hospital. The characteristics (sex, age) of participants are in line with other Dutch studies [8, 25]. Nevertheless, we cannot exclude with certainty that patient selection was fully absent.

Patients in this study came from three hospitals in a region where prehospital and hospital healthcare providers have regular meetings to optimize care for patients with acute coronary syndrome and stroke. These networks exist throughout the Netherlands where similar guidelines are used. Therefore, our results are probably applicable throughout the country. In other countries organization of emergency medicine may differ [26], resulting into various other paths and time intervals.

## Conclusions

Overall, efforts to reduce patient delay may lead to a relatively higher reduction in time between onset of symptoms and arrival in hospital than efforts to reduce delays involving health care providers. However, since reducing patient delay is difficult, and noticeable differences do exist between various prehospital paths where often two or more health care providers are involved, further research into reasons for these differences may yield possibilities to optimize paths and thus reduce overall time delay. Evidence is appearing that in the Dutch situation it is better, as it is in many other countries around the world, to bypass the GP in case of ACS or stroke and directly call the national emergency number.

## Abbreviations

ACS: acute coronary syndrome
CCU: coronary care unit
ECG: electrocardiogram
ED: emergency department
GP: general practitioner
IQR: interquartile ranges
MI: myocardial infarction
PCI: percutaneous coronary intervention
rt-PA: recombinant tissue plasminogen activator
SD: standard deviation

## References

[1] The Task Force on the management of ST-segemtn elevation acute moycardial infarcton of the European Society of Cardiology (ESC) (2012). ESC Guidelines for the management of acute myocardial infarction in patients presenting with ST-segment elevation. Eur Heart J.

[2] Jauch EC, Saver JL, Adams HP, Bruno A, Connors JJ, Demaerschalk BM (2013). Guidelines for the early management of patients with acute ischemic stroke: a guideline for healthcare professionals from the American Heart Association/American Stroke Association. Stroke.

[3] Lees KR, Bluhmki E, von Kummer R, Brott TG, Toni D, Grotta JC (2010). Time to treatment with intravenous alteplase and outcome in stroke: an updated pooled analysis of ECASS, ATLANTIS, NINDS, and EPITHET trials. Lancet.

[4] Fassbender K, Balucani C, Walter S, Levine SR, Haass A, Grotta J (2013). Streamlining of prehospital stroke management: the golden hour. Lancet Neurol.

[5] Evenson KR, Foraker RE, Morris DL, Rosamond WD (2009). A comprehensive review of prehospital and in-hospital delay times in acute stroke care. Int J Stroke.

[6] Herlitz J, Wireklintsundstrom B, Bang A, Berglund A, Svensson L, Blomstrand C (2010). Early identification and delay to treatment in myocardial infarction and stroke: differences and similarities. Scand J Trauma Resusc Emerg Med.

[7] Bray JE, Straney L, Barger B, Finn J (2015). Effect of public awareness campaigns on calls to ambulance across Australia. Stroke.

[8] Meijer RJ, Hilkemeijer JH, Koudstaal PJ, Dippel DW (2004). Modifiable determinants of delayed hospital admission following a cerebrovascular accident. Ned Tijdschr Geneeskd.

[9] Price CI, Rae V, Duckett J, Wood R, Gray J, McMeekin P (2013). An observational study of patient characteristics associated with the mode of admission to acute stroke services in North East England. PLoS One.

[10] Buck BH, Starkman S, Eckstein M, Kidwell CS, Haines J, Huang R (2009). Dispatcher recognition of stroke using the National Academy Medical Priority Dispatch System. Stroke.

[11] Jones SP, Carter B, Ford GA, Gibson JM, Leathley MJ, McAdam JJ (2013). The identification of acute stroke: an analysis of emergency calls. Int J Stroke.

[12] Caceres JA, Adil MM, Jadhav V, Chaudhry SA, Pawar S, Rodriguez GJ (2013). Diagnosis of stroke by emergency medical dispatchers and its impact on the prehospital care of patients. J Stroke Cerebrovasc.

[13] Watkins CL, Leathley MJ, Jones SP, Ford GA, Quinn T, Sutton CJ (2013). Training emergency services’ dispatchers to recognise stroke: an interrupted time-series analysis. BMC Health Serv Res.

[14] Saver JL, Smith EE, Fonarow GC, Reeves MJ, Zhao X, Olson DM (2010). The “golden hour” and acute brain ischemia: presenting features and lytic therapy in >30,000 patients arriving within 60 minutes of stroke onset. Stroke.

[15] Chen W, Woods SL, Puntillo KA (2005). Gender differences in symptoms associated with acute myocardial infarction: a review of the research. Heart Lung.

[16] Alconero-Camarero AR, Munoz-Cacho P, Revuelta JM (2013). Gender similarities and differences in the presentation of symptoms in acute myocardial infarction. Int J Cardiol.

[17] Stuart-Shor EM, Wellenius GA, DelloIacono DM, Mittleman MA (2009). Gender differences in presenting and prodromal stroke symptoms. Stroke.

[18] Moser DK, Kimble LP, Alberts MJ, Alonzo A, Croft JB, Dracup K (2006). Reducing delay in seeking treatment by patients with acute coronary syndrome and stroke: a scientific statement from the American Heart Association Council on cardiovascular nursing and stroke council. Circulation.

[19] Chang KC, Tseng MC, Tan TY (2004). Prehospital delay after acute stroke in Kaohsiung, Taiwan. Stroke.

[20] Mandelzweig L, Goldbourt U, Boyko V, Tanne D (2006). Perceptual, social, and behavioral factors associated with delays in seeking medical care in patients with symptoms of acute stroke. Stroke.

[21] Mosley I, Nicol M, Donnan G, Patrick I, Dewey H (2007). Stroke symptoms and the decision to call for an ambulance. Stroke.

[22] Caldwell MA, Miaskowski C (2002). Mass media interventions to reduce help-seeking delay in people with symptoms of acute myocardial infarction: time for a new approach?. Patient Educ Couns.

[23] Lecouturier J, Rodgers H, Murtagh MJ, White M, Ford GA, Thomson RG (2010). Systematic review of mass media interventions designed to improve public recognition of stroke symptoms, emergency response and early treatment. BMC Public Health.

[24] Reeves MJ (2012). Reducing the delay between stroke onset and hospital arrival: is it an achievable goal?. JAHA.

[25] Lahr MM, Luijckx GJ, Vroomen PC, van der Zee DJ, Buskens E (2012). Proportion of patients treated with thrombolysis in a centralized versus a decentralized acute stroke care setting. Stroke.

[26] Fleischmann T, Fulde G (2007). Emergency medicine in modern Europe. Emerg Med Australas.

